# Patterns and Clinical Predictors of Antiseizure Medication Use in Pediatric Traumatic Brain Injury

**DOI:** 10.21203/rs.3.rs-10648062/v1

**Published:** 2026-08-26

**Authors:** Deena S Godfrey, Marta Fernandes, Kevin J Staley, Ann-Christine Duhaime, Sahar F Zafar

**Affiliations:** Massachusetts General Hospital; Massachusetts General Hospital; Massachusetts General Hospital; Massachusetts General Hospital; Massachusetts General Hospital

**Keywords:** Traumatic brain injury, seizures, antiseizure medication, pediatric neurocritical care

## Abstract

**Objective:**

Current guidelines recommend the use of antiseizure medications (ASM) in pediatric patients with severe traumatic brain injury (TBI). This is based on low-quality evidence and can lead to variability in practice and over-prescription of medications. To understand factors influencing ASM prescription in pediatric TBI, we examined clinical variables associated with inpatient and discharge ASM prescription.

**Methods:**

Multi-center, retrospective cohort study of patients aged 0 to 21 years, admitted with TBI to the intensive care units at two Level I trauma centers, from 2016 through 2024. Obtained demographic and clinical information from chart review. Neuroimaging and electroencephalogram findings were extracted from reports. Primary outcome measure was ASM administration during inpatient hospitalization. Secondary outcome measure was prescription of ASMs at discharge. We developed multivariable logistic regression models to identify factors associated with inpatient and discharge ASM prescription, and report adjusted odds ratios (OR) with 95% confidence intervals (CI).

**Results:**

467 patients met inclusion criteria. 287 (61%) patients were given an ASM during their hospitalization, and 159 (34%) patients were discharged from the hospital on an ASM. Factors associated with higher likelihood of inpatient ASM prescription include intracranial injury on imaging (OR 57, 95% CI 24–156), moderate Glasgow Coma Scale score of 9 to 12 (OR 21, 95% CI 2.4–354), neurology/neurosurgery admission (OR 11, 95% CI 3.1–42), and immediate seizures (OR 3.6, 95% CI 0.77-23). Clinical factors associated with greater likelihood of discharge ASM prescription were neuroimaging abnormalities (OR 8.5, 95% CI 4.0–20), immediate seizures (OR 6.1, 95% CI 1.9–22), and neurology/neurosurgery admission (OR 2.1, 95% CI 0.71–6.8).

**Conclusions:**

Neuroimaging abnormalities, seizures, and admission to neurology or neurosurgery services were associated with greater likelihood of ASM prescription during hospitalization and at discharge. Future studies are needed to determine whether ASM prescription improves outcomes and optimize ASM use.

## Introduction

Traumatic brain injury (TBI) is associated with significant morbidity and mortality in children^1^. Early post-traumatic seizures (PTS) occur within the first 7 days after a TBI and can contribute to secondary injury by causing increased metabolic demands, alterations in cerebral blood flow, elevated intracranial pressure, and abnormal neurotransmitter activity^2,3^.

To reduce the risk of secondary brain injury and attempt to prevent early PTS, prophylactic antiseizure medications (ASMs) are frequently used in the acute phase of TBI^4–13^. There is significant variability in ASM prophylaxis practices (e.g., ASM choice, patient selection, duration) for patients with severe TBI^12–16^. In an attempt to standardize this practice, the Brain Trauma Foundation released updated guidelines in 2019 for severe pediatric TBI, recommending a 7-day course of seizure prophylaxis (level III, low quality of evidence), with insufficient evidence to recommend levetiracetam over phenytoin^17^. Adult guidelines are also limited by low quality of evidence. In 2024, The Neurocritical Care Society published guidelines for seizure prophylaxis in adults with moderate-severe TBI^18^. Based on GRADE criteria, they presented weak recommendations (low quality of evidence) for the use of ASMs as prophylaxis in this population^18^. Levetiracetam was recommended rather than phenytoin/fosphenytoin, but again this was a weak recommendation (very low quality of evidence)^18^. The low quality of evidence, particularly for pediatric populations, results in lack of consensus and practice variations in the field leading to variance in outcomes^19^. To ensure that pediatric patients at risk for early post-traumatic seizures are prescribed ASMs for appropriate indications and duration, it is imperative to first understand the state of current practice of ASM prescription in this population.

In this study, we evaluated ASM prescription practices over an 8-year period. We sought to examine clinical variables (including injury type, etiology, severity and electroencephalogram use) associated with 1) inpatient ASM prescription, and 2) continued ASM prescription at discharge. We additionally sought to evaluate differences in prescription patterns after publication of the 2019 Brain Trauma Foundation guidelines.

## Methods

### Study design

This is a multi-center, retrospective cohort study of patients aged 0 to 21 years, admitted with TBI to the intensive care units (ICUs) at two Level I trauma centers (Massachusetts General Hospital, Brigham and Women’s Hospital) from January 1, 2016 through December 31, 2024. The protocol was approved by the Institutional Review Board of Mass General Brigham (Protocol number 2025P000467). Informed consent was not required for this study.

We included patients with, (1) age ≤ 21 years; (2) admission for traumatic brain injury (as defined by International Classification of Diagnoses Version 10, ICD-10); (3) Glasgow Coma Scale (GCS) score of 3 to 8 and/or admission to an intensive care unit.

Validated ICD-10 codes for accurately identifying TBI in pediatric^20–22^ and adult^23–25^ populations were used to identify patients with TBI. ICD codes included S02 (traumatic fractures of the skull and facial bones), S06 (intracranial injuries), S07 (crushing injuries of the head), S09.7 (multiple injuries of the head), S09.8 (other specified injuries of the head), S09.9 (unspecified injury of face and head).

### Clinical covariates

Detailed chart review was performed for included patients and the following clinical information was obtained: patient demographics, past medical history, dates of admission for acute TBI, length of stay in ICU, hospital length of stay, admitting service (neurology, neurosurgery, medical, and surgical specialties), GCS scores in the first 24 hours, mechanism of injury, neuroimaging findings, seizure incidence (within first 24 hours and/or during hospital admission), electroencephalogram findings, administration of ASMs, ASM agents used, duration of ASM treatment, discharge ASM prescription and duration, and discharge disposition. TBI severity was defined using the worst GCS score within the first 24 hours of injury^26^. Mild TBI was defined as GCS score 13 to 15, moderate as GCS score 9 to 12, and severe as GCS score 3 to 8. Children with mild and moderate GCS scores who were admitted to the intensive care unit (ICU) were included in this study because GCS scores can be inconsistent^27,28^, particularly in pediatric patients, and if ICU-level of care was indicated, we assumed that these injuries were more serious.

Mechanisms of injury included gunshot wounds, assault, fall, motor-vehicle-accidents (including pedestrians or cyclists struck by vehicles), non-accidental trauma, and struck by/against objects^29^.

ASM use for more than 24 hours was recorded and the ASMs of interest included: levetiracetam, valproic acid, lacosamide, phenytoin/fosphenytoin, lamotrigine, carbamazepine, oxcarbazepine, clobazam, topiramate, and zonisamide. While general management protocols were in place for treatment of patients with severe TBI requiring ICU care, many of these allowed for clinician judgement so there was variability among treating clinicians about how care was managed with respect to ASM administration.

Neurosurgical procedures included the placement of intracranial pressure monitors, external ventricular drains, subdural drains, burr holes, and shunts. In addition, surgical procedures such as fracture repairs, craniotomies, and craniectomies were included.

### Physiologic data

For patients with electroencephalogram (EEG) data, individual reports during hospitalizations were reviewed and the abnormalities were classified based on the American Clinical Neurophysiology Society’s (ACNS) Standardized Critical Care EEG Terminology^30^. The ACNS categorizes epileptiform findings as sporadic epileptiform discharges and rhythmic and periodic patterns^30^. The rhythmic and periodic patterns are further subdivided into two terms: Main term 1 (generalized, lateralized, bilateral independent, unilateral independent, multifocal) and Main term 2 (periodic discharges, rhythmic delta activity, spike and wave, polyspike and wave, sharp and wave)^30^. The following abnormalities, if present, were recorded: lateralized rhythmic delta activity (LRDA), generalized rhythmic delta activity (GRDA), generalized periodic discharges (GPD), lateralized periodic discharges (LPD), sharps, epileptiform discharges, spike and wave complexes, burst suppression, and seizures. For use in multivariable logistic regression models, EEG results were categorized as normal, abnormalities (LRDA, GRDA, GPD, LPD, sharps, discharges, spike and wave complexes, burst suppression), and seizures.

### Neuroimaging

For patients with neuroimaging data, either from Computed Tomography (CT) or Magnetic Resonance Imaging (MRI), individual imaging reports were reviewed and abnormalities were classified as follows: subdural hemorrhage, subarachnoid hemorrhage, epidural hemorrhage, intraventricular hemorrhage, intraparenchymal hemorrhage, skull fractures, diffuse axonal injury, herniation, cerebral edema, contusions, hydrocephalus, and anoxic/hypoxic/ischemic brain injury. When imaging was incorporated into modeling, imaging was divided into “normal” and “any intracranial abnormality” (including subdural hemorrhage, subarachnoid hemorrhage, epidural hemorrhage, diffuse axonal injury, and intraparenchymal hemorrhage).

### Outcomes

Our primary outcome measure was ASM administration during inpatient hospitalization. Our secondary outcome measure was prescription of ASMs at discharge.

### Statistical analysis

Clinical variables were derived from the electronic health record and neurodiagnostic data. Composite variables were created for the presence of any intracranial injury (subdural hemorrhage, subarachnoid hemorrhage, epidural hemorrhage, diffuse axonal injury, or intraparenchymal hemorrhage) and for any EEG abnormality (LRDA, GRDA, GPD, LPD, sharps, discharges, spike and wave complexes, burst suppression). Binary variables were coded as categorical factors. Missing data were retained as separate “Unknown” categories.

Baseline characteristics were summarized according to ASM use during hospitalization. Continuous variables were reported as median (Quartile 1, Quartile 3) and categorical variables as counts and percentages. Group comparisons were performed between patients receiving ASM during hospitalization or discharge versus those that did not.

The primary outcome was initiation of an ASM during hospitalization. The dataset was randomized with respect to year and was randomly divided into training (70%) and testing (30%) cohorts using stratified sampling to preserve the prevalence of ASM use. Model development was performed exclusively in the training cohort, while model performance was evaluated in the independent testing cohort. The secondary outcome was prescription of ASMs at discharge. When modeling patients prescribed an ASM at discharge, those who died during their hospitalization were excluded from the discharge ASM analysis.

A multivariable logistic regression model was developed to identify factors associated with inpatient ASM administration. The variables included in the model were selected a priori based on clinical relevance and previously published literature regarding factors associated with early post-traumatic seizures. These predictor variables included age, sex, admitting service, traumatic brain injury severity categorized by highest GCS score in the first 24-hours after injury, mechanism of injury, presence of intracranial injury on neuroimaging, abnormalities on EEG, occurrence of immediate post-traumatic seizures (within the first 24-hours after injury), and need for neurosurgical procedures. Adjusted odds ratios (ORs) with 95% confidence intervals (CIs) were calculated by exponentiating model coefficients.

To evaluate model stability and internal validity, 5-fold cross-validation was performed within the training cohort. Cross-validated discrimination was assessed using the area under the receiver operating characteristic curve (ROC-AUC).

Predicted probabilities were generated for both the training and testing cohorts. Calibration was performed using Platt scaling, which fits a secondary logistic regression model to the predicted probabilities in the training cohort and applies the resulting calibration model to the testing cohort. Calibration performance was evaluated visually using calibration plots comparing predicted and observed event rates across deciles of risk and quantitatively using calibration statistics implemented in the rms package.

Model discrimination was assessed in the testing cohort using receiver operating characteristic (ROC) analysis and the area under the ROC curve (ROC-AUC). An optimal probability threshold for classification was determined in the training cohort using the Youden index and subsequently applied to the testing cohort. Classification performance was summarized using confusion matrices and reported as sensitivity, specificity, positive predictive value (PPV), negative predictive value (NPV), and accuracy.

All analyses were performed in RStudio Version 2026.05.1. A two-sided p-value < 0.05 was considered statistically significant.

### Subgroup analysis

In addition to evaluating which patients were prescribed ASMs during TBI hospitalizations and at time of discharge, we also evaluated whether there was a change in ASM prescription practices with publication of the 2019 Brain Trauma Foundation Guidelines. The Brain Trauma Foundations Guidelines were published on March 31, 2019^17^. To account for distribution and implementation of the guidelines, we chose Jan 1, 2020 as a reasonable timepoint by which to determine if there were changes in ASM prescription for early seizure prophylaxis in the pediatric TBI population.

## Results

### Cohort Characteristics

**Figure 1:**
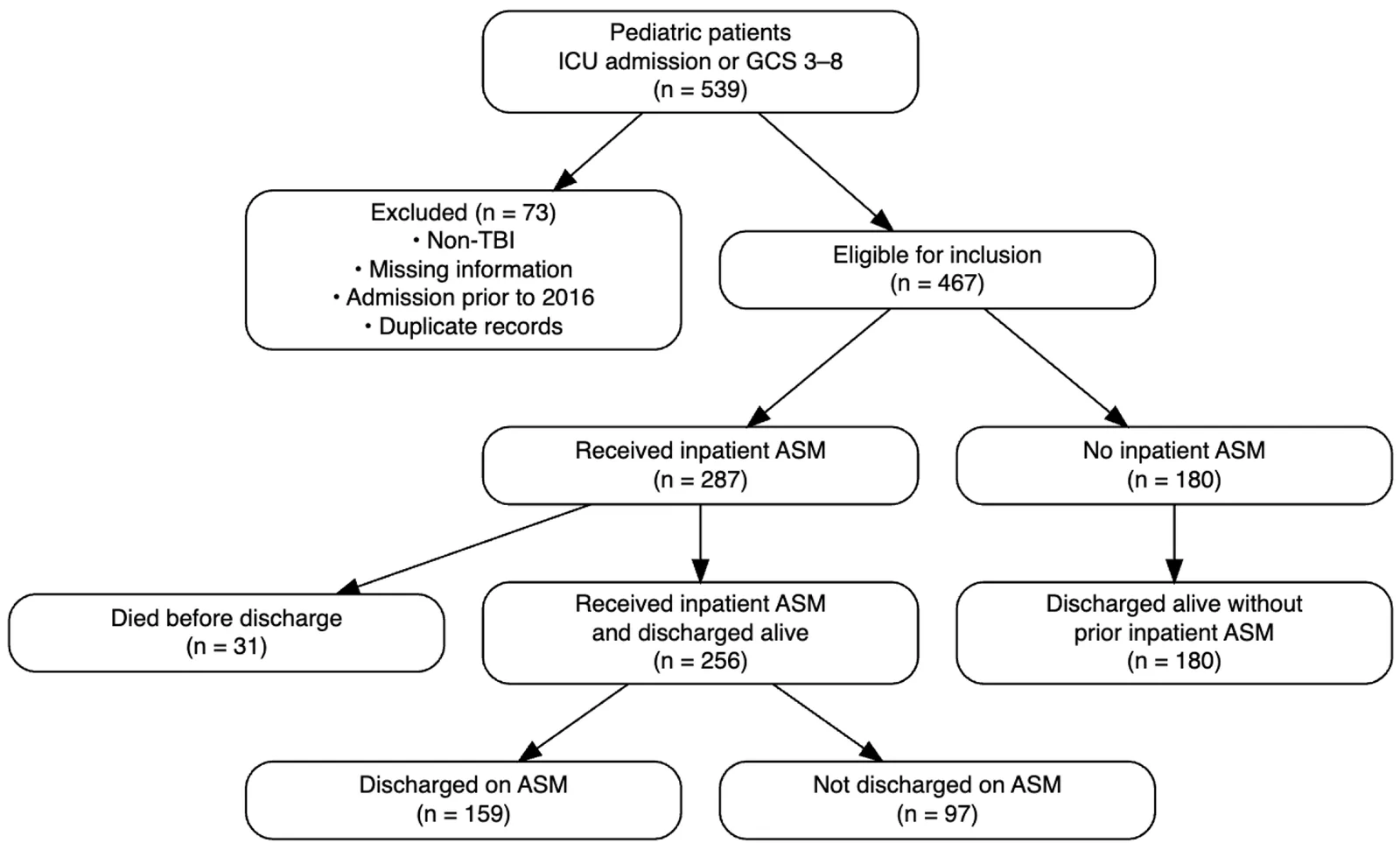
Diagram of Exclusion Criteria and Number of Patients in the Analysis GCS: Glasgow Coma Scale, ICU: intensive care unit, TBI: traumatic brain injury

Demographic data for the complete pediatric ICU cohort is summarized in Table 1. Our final cohort comprised 467 pediatric patients admitted to an ICU with a TBI. Most patients were male (n=335, 72%), white (n=262, 56%), and non-Hispanic (n=288, 75%) (Table 1). The median age was 16 years old (8-19), and most injuries were categorized as mild (n=293, 67%) (GCS of 13-15). The most common mechanisms of injury were motor vehicle collisions (MVC, n=221, 47%), falls (n=173, 37%), and assaults (n=19, 4.1%). The overall inpatient mortality rate was 6.6% (n=31), leading to a final discharge patient cohort of 436 patients (Figure 1).

### Primary Outcome: Inpatient ASM Administration

Demographic and clinical features of pediatric ICU patients prescribed an ASM during a TBI hospitalization are summarized in Table 1. Two hundred and eighty-seven patients (66%) were prescribed ASMs during their hospitalization. Similarly to the complete pediatric ICU cohort, the majority of patients prescribed an inpatient ASM were white (n=162, 56%), non-Hispanic (n=174, 75%), with a median age of 17 years (9, 20).

**Figure 2:**
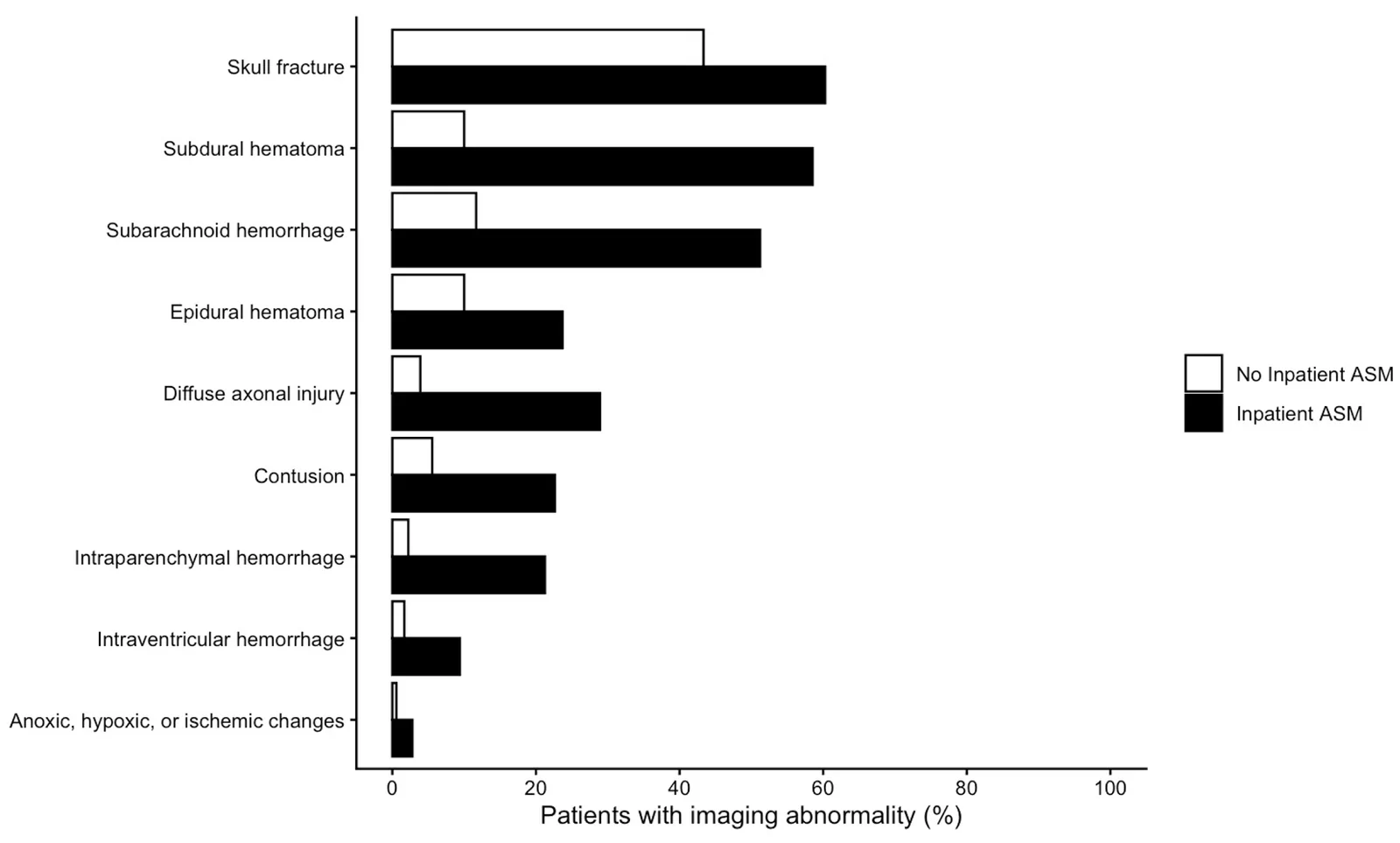
Neuroimaging Abnormalities of Patients Prescribed an Inpatient ASM Compared to Those Not Prescribed an ASM ASM: antiseizure medication

**Figure 3:**
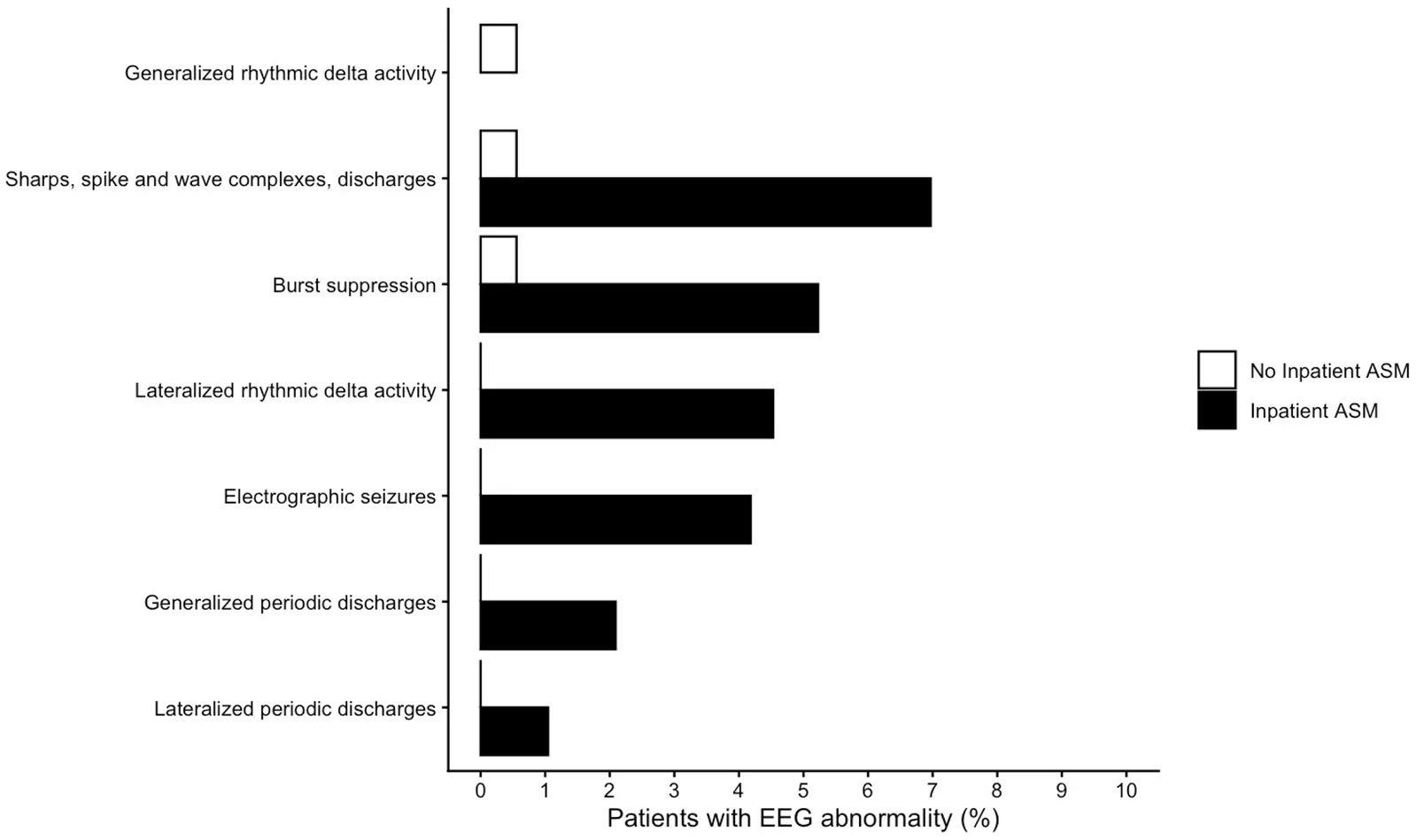
EEG Abnormalities of Patients Prescribed an Inpatient ASM Compared to Those Not Prescribed an ASM ASM: antiseizure medication; EEG: electroencephalogram

When comparing patients prescribed an ASM during their hospitalization to those who were not, those prescribed an ASM were older (median age of 17 compared to 14, p=0.002) and male (76% compared to 66%, p=0.02). Those prescribed an ASM were more likely to have moderate and severe TBIs, be admitted to a neurology or neurosurgical service, undergo a neurosurgical procedure, and have longer lengths of stay (6 versus 3 days). The various imaging and electrographic abnormalities seen across the inpatient ASM versus no inpatient ASM cohorts are presented in Figure 2 and 3. There were significant differences between the groups as to discharge disposition, with the cohort that did not receive ASMs primarily discharged home (n=139, 77%) compared to the inpatient ASM cohort (n=154, 54%).

Those prescribed an ASM during inpatient admission had a median course of 5 days of ASM prescription (IQR: 2-7). Levetiracetam was the most prescribed ASM, followed by valproic acid (Supplemental Figure 1). Patients prescribed an inpatient ASM were significantly more likely to have had an immediate seizure at the time of injury (12% versus 1.7%, p<0.001) and to have had a subsequent seizure during admission (3.1% versus 0%, p=0.015).

Of note, 41 subjects (14%) with no seizures (immediate or subsequent) were prescribed ASMs for greater than 7 days. Mean duration of ASM prescription was 20 days, with a range of 8 days to 69 days. Within this subgroup, 21 (51%) patients had a GCS of 3 to 8 on admission and 22 (54%) were admitted to the neurosurgical service. All these patients had abnormal neuroimaging (subdural hemorrhage= 28, 68%; subarachnoid hemorrhage = 24, 59%; intraparenchymal hemorrhage = 17, 41%) and 32 (63%) underwent a neurosurgical procedure during their admission.

**Figure 4:**
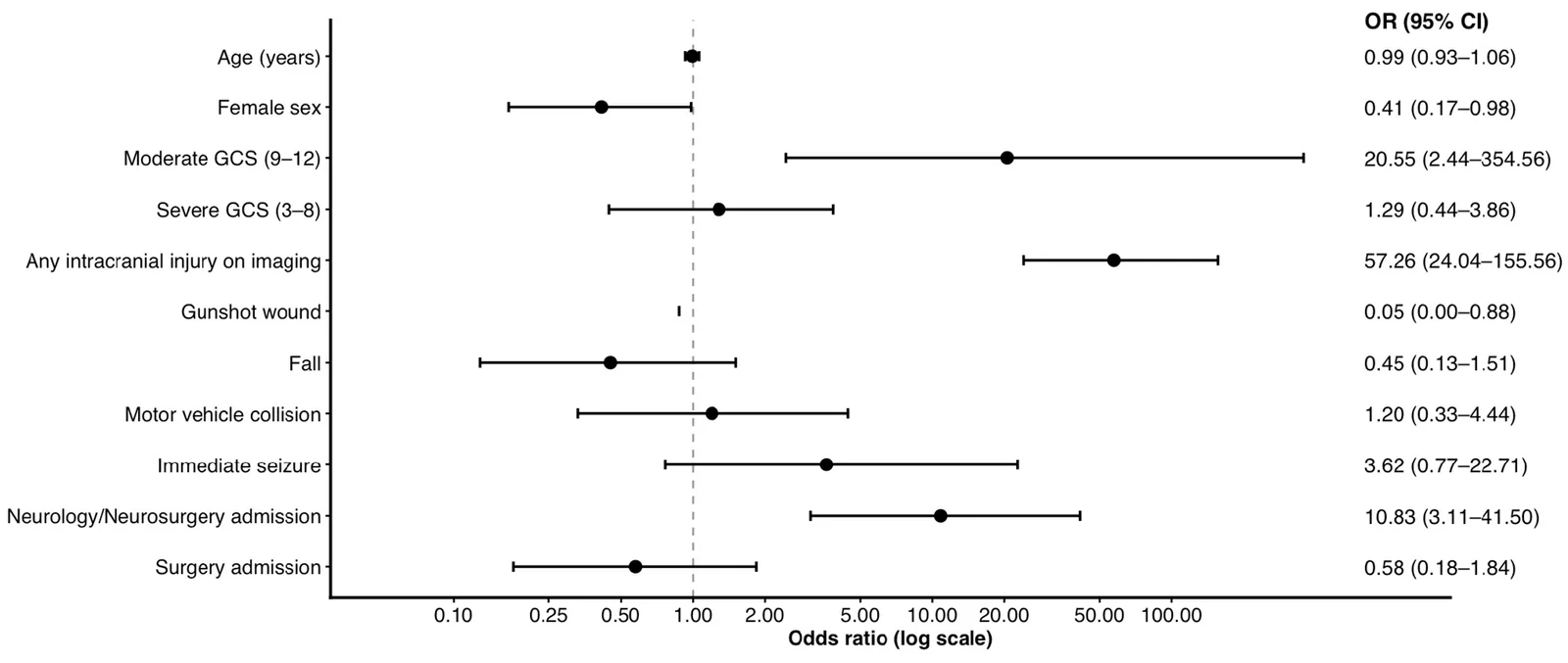
Factors Associated with Inpatient ASM Prescription in All Pediatric TBI Patients ASM: antiseizure medication, GCS: Glasgow Coma Scale, TBI: traumatic brain injury

**Figure 5:**
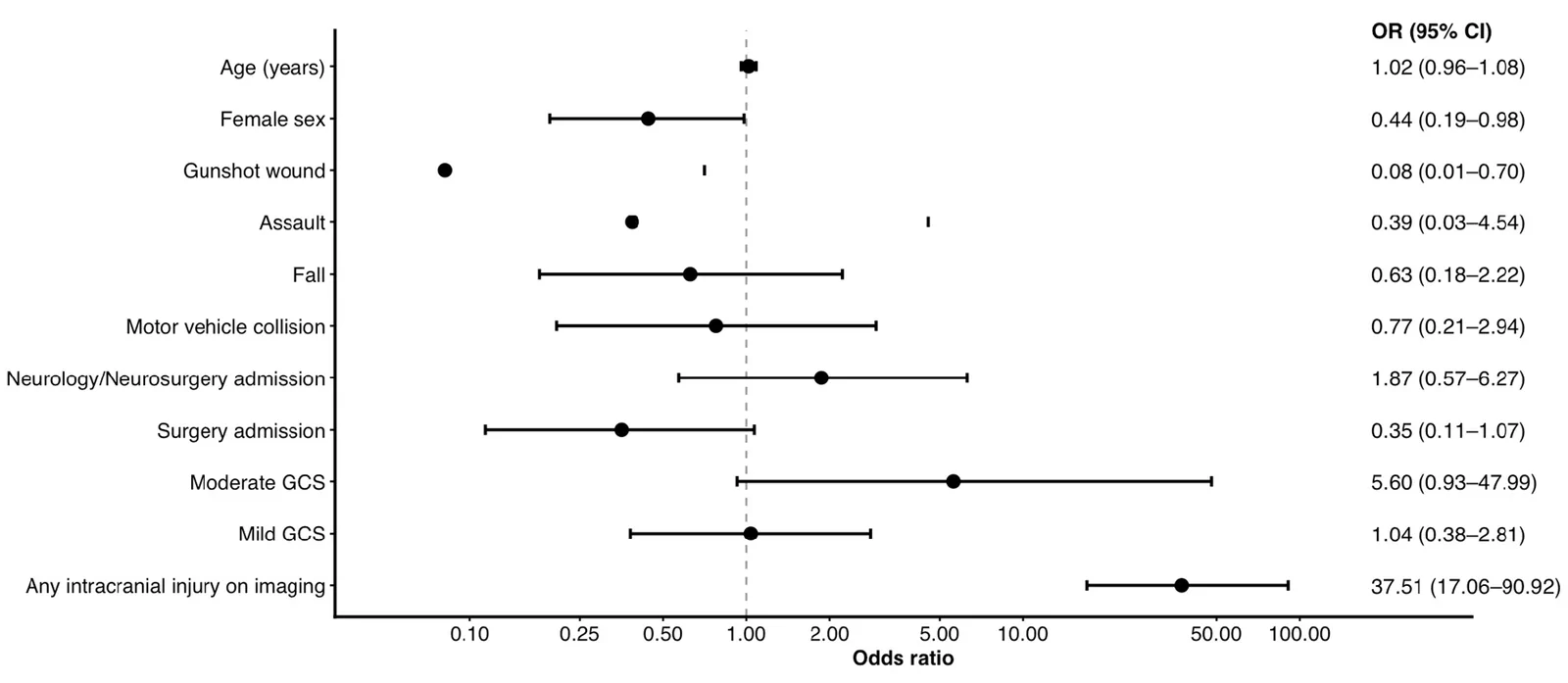
Factors Associated with Inpatient ASM Prescription in All Pediatric TBI Patients Without Immediate Seizures ASM: antiseizure medication, GCS: Glasgow Coma Scale, TBI: traumatic brain injury

To determine whether immediate seizures impacted the likelihood of being prescribed an ASM while inpatient, logistic regression models were developed for all patients prescribed ASMs (including those with immediate seizures, Figure 4) and for a subset of patients with no immediate seizures (Figure 5). Factors associated with higher likelihood of inpatient ASM prescription include intracranial injury on imaging (OR 57, 95% CI 24-156), moderate GCS of 9 to 12 (OR 21, 95% CI 2.4-354), neurology/neurosurgery admission (OR 11, 95% CI 3.1-42), and immediate seizures (OR 3.6, 95% CI 0.77-23) (Figure 4). After excluding patients with immediate seizures, neuroimaging abnormalities on imaging (OR 38, 95% CI 17-91), moderate GCS (OR 5.6, 95% CI 0.93-48), and neurology/neurosurgery admission (OR 1.9, 95% CI 0.57-6.3) were associated with greater odds of being prescribed an ASM during inpatient admission.

### Secondary Outcome: Discharge ASM Prescription

**Figure 6:**
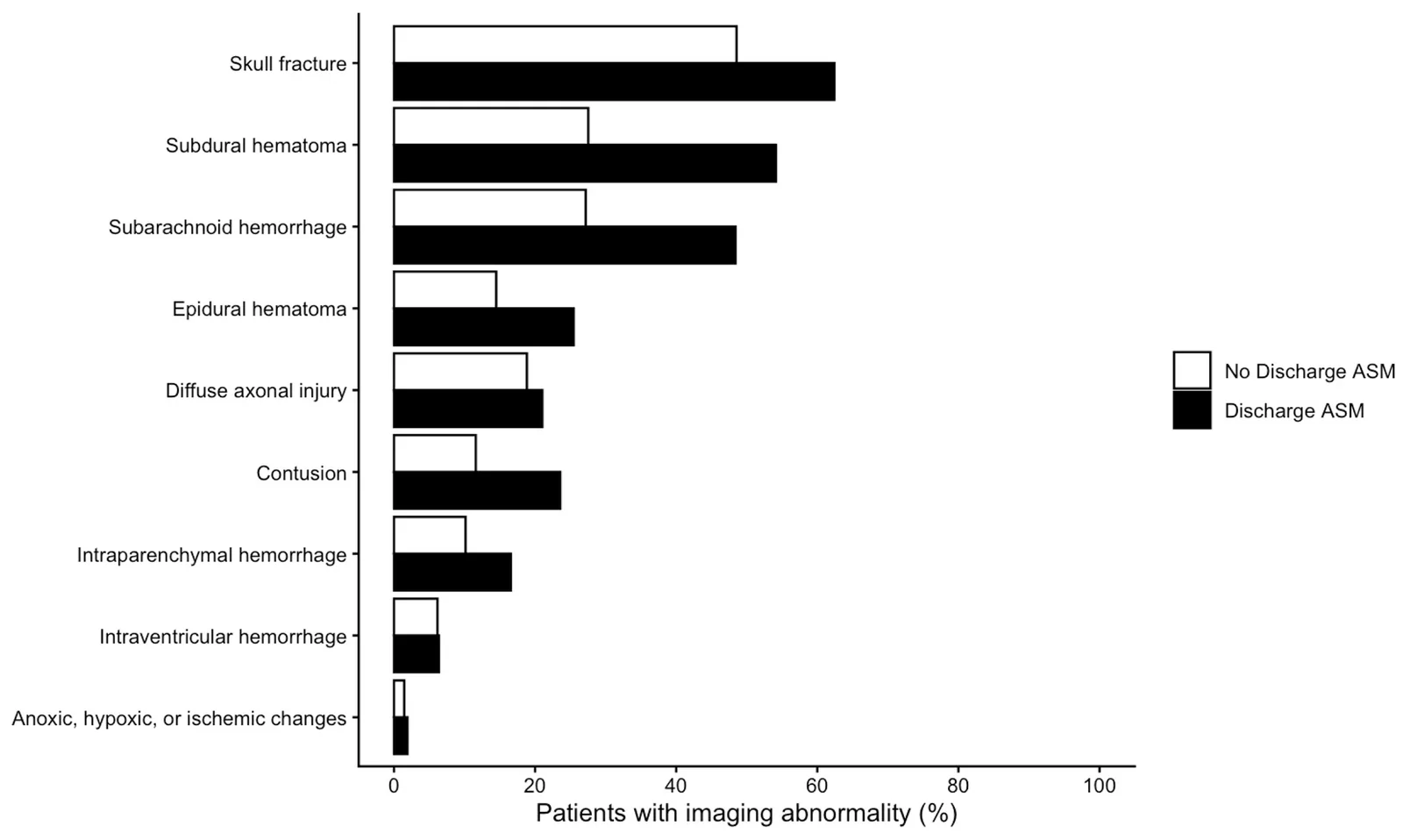
Neuroimaging Abnormalities of Patients Prescribed a Discharge ASM Compared to Those Not Prescribed an ASM ASM: antiseizure medication

**Figure 7:**
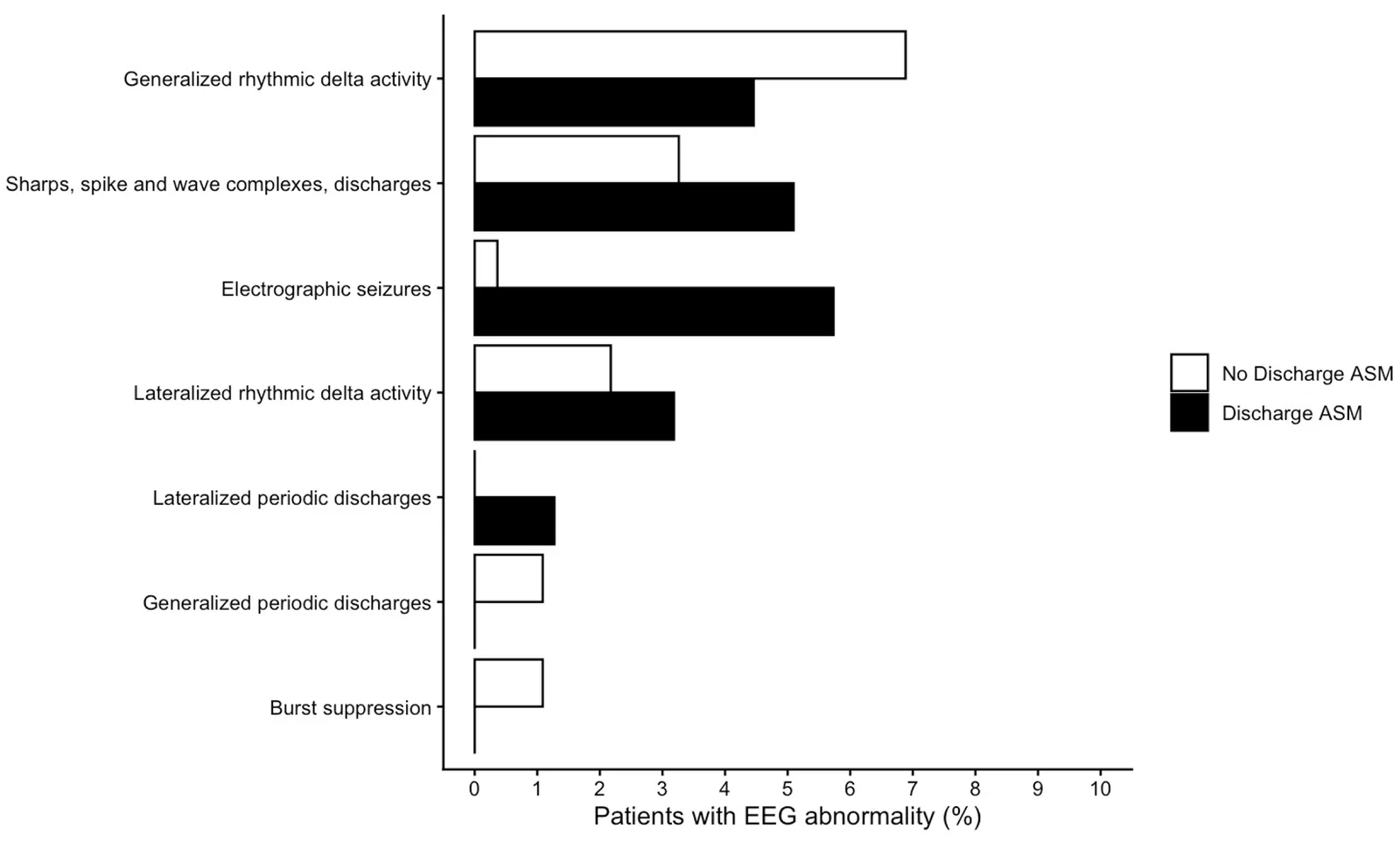
EEG Abnormalities of Patients Prescribed a Discharge ASM Compared to Those Not Prescribed an ASM ASM: antiseizure medication, EEG: electroencephalogram

Of the entire pediatric ICU cohort, there were 31 (6.6%) patients who expired during their TBI hospitalization. These patients were removed from the analysis of factors associated with discharge ASM prescription (Figure 1). Demographic and clinical features are summarized in Table 2. A total of 159 (36%) patients were discharged on an ASM (these patients had also been administered an ASM while inpatient). Compared to the inpatient ASM cohort, there was a similar demographic breakdown with regards to age, sex, race, and ethnicity. Those discharged on an ASM were more likely to be admitted to a neurology service (7.1% versus 1.8%) or a neurosurgical service (41% versus 23%) (p<0.001). Of the patients prescribed an ASM at discharge, 107 (73%) had a mild GCS, 19 (13%) had a moderate GCS, and 20 (14%) had a severe GCS at admission. Patients who were not prescribed an ASM at discharge were more likely to have a severe GCS at admission (23% versus 14%, p=0.01). The specific imaging and electrographic abnormalities seen across the discharge ASM versus no discharge ASM cohorts are presented in Figures 6 and 7.

Those patients discharged on an ASM were typically prescribed a 7-day course (median 7 days, IQR 7-7) with a range from 5 days to 30 days. 49 patients were not given a clear end-date of their ASM prescription at discharge. Those discharged on an ASM were significantly more likely to have had an immediate seizure at time of injury (15% versus 4%, p<0.001) and a subsequent seizure during their hospitalization (3.8% versus 0.4%, p=0.01), but no major differences were found when looking at discharge ASM among patients requiring neurosurgical procedures.

**Figure 8:**
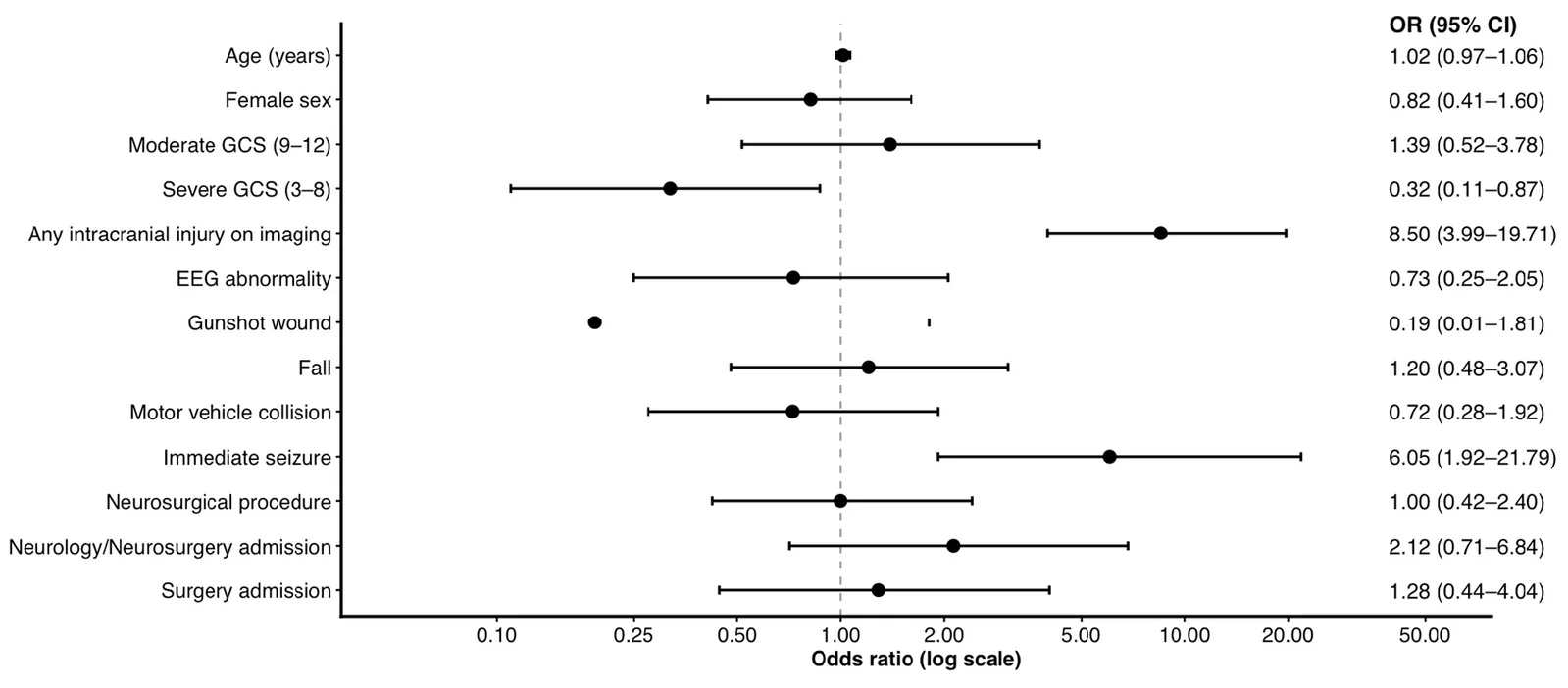
Factors Associated with Discharge ASM Prescription in All Pediatric TBI Patients ASM: antiseizure medication, GCS: Glasgow Coma Scale, TBI: traumatic brain injury

**Figure 9:**
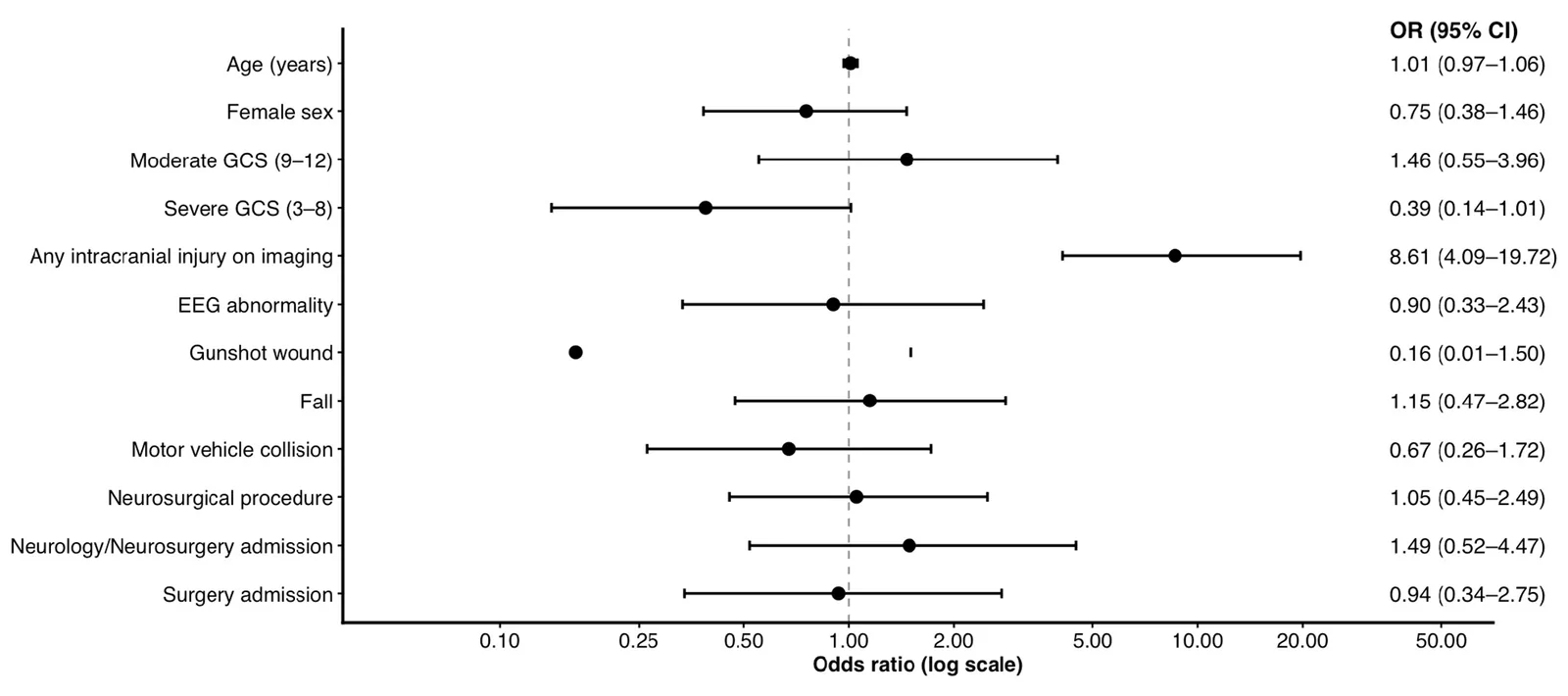
Factors Associated with Discharge ASM Prescription in Pediatric TBI Patients Without Immediate Seizures ASM: antiseizure medication, GCS: Glasgow Coma Scale, TBI: traumatic brain injury

The factors associated with discharge ASM prescription among the entire discharge cohort (including those with immediate seizures) are presented in Figure 8. Clinical factors associated with greater likelihood of discharge ASM prescription were neuroimaging abnormalities (OR 8.5, 95% CI 4.0-20), immediate seizures (OR 6.1, 95% CI 1.9-22), and neurology/neurosurgery admission (OR 2.1, 95% CI 0.71-6.8). When patients with seizures were removed from the model (Figure 9), presence of neuroimaging abnormalities was the predominant factor associated with discharge ASM prescription (OR 8.6, 95% CI 4.1-19.7).

### Predictive Model Performance

The predictive models demonstrated good discrimination for both inpatient and discharge ASM prescribing (Supplemental Table 2). For inpatient ASM prescription, the model achieved an area under the receiver operating characteristic curve (ROC-AUC) of 0.76 (95% CI 0.67-0.85), which improved to 0.84 (95% CI 0.77-0.91) when restricted to patients without immediate seizures. For discharge ASM prescription, the model demonstrated an AUC of 0.83 (95% CI 0.75-0.91) in the full cohort and 0.79 (95% CI 0.70-0.88) among patients without immediate seizures. The model had higher negative predictive values than positive predictive values for discharge ASM prescription (87.9%–90.0% vs. 55.3%–59.7%), indicating better performance in identifying patients who were not discharged on ASMs than those who were.

### Subgroup analysis

When comparing ASM prescription practices prior to and including Dec 31, 2019 to after Jan 1, 2020, levetiracetam was most frequently prescribed both during inpatient admission and at time of discharge. There were no significant differences in ASM agent selection when comparing patients admitted from 2016-2019 to 2020-2024 (Supplemental Table 1).

## Discussion

In this retrospective cohort study, we found that in pediatric patients admitted to an ICU with a TBI, those prescribed an ASM during their hospital admission and at discharge were significantly more likely to have moderate/severe injuries, be admitted to a neurology or neurosurgical service, have longer lengths of stay, and abnormal findings on imaging and EEG. All patients prescribed an ASM at hospital discharge had received ASMs during their admission. Logistic regression models of likelihood of inpatient ASM and discharge ASM prescription were performed. Variables associated with greater likelihood of inpatient and discharge ASM prescription include intracranial injury on imaging, moderate GCS, neurology/neurosurgery admission, and immediate seizures. We did not find any significant difference in which ASM agent was chosen when comparing prescription practices before and after publication of the Brain Trauma Foundation 2019 guidelines.

In this study, admission to a neurology and neurosurgery service was associated with a greater likelihood of being prescribed an ASM during admission and at time of discharge. It is possible that these patients had greater levels of injury or neurological symptoms, leading to admission on a neuroscience floor compared to surgical or medical specialties. Alternatively, clinical and specialty-specific differences in prescribing practices may contribute to the observed association. A fair percentage of patients with a mild GCS were prescribed an ASM during inpatient admission and at time of discharge, which is outside the recommendations from the Brain Trauma Foundation which prioritizes ASM prophylaxis for severe TBI^17^. Consistent with previous retrospective studies in pediatric^13,31,32^ and adult populations^33,34^, a substantial proportion of patients with mild and moderate TBI in our cohort were prescribed ASMs possibly relating to imaging abnormalities or other clinical markers of injury severity. This finding highlights the continued variability in seizure prophylaxis practices and may reflect the challenges clinicians face in identifying which patients are most likely to benefit from ASM therapy.

Although not statistically significant, patients discharged with an ASM had shorter hospital admissions than those not prescribed an ASM at discharge (4 vs. 7 days). This finding may reflect completion of seizure prophylaxis during hospitalization among patients with longer admissions, thereby reducing the need for discharge ASM prescriptions. Similarly, patients with severe TBI (GCS 3–8) were less likely to be discharged on an ASM than those with a mild initial GCS. Given that severe TBI is frequently associated with prolonged hospitalization, these patients may have completed their intended prophylactic ASM course prior to discharge, resulting in lower rates of discharge ASM use. Additionally, newer recommendations for characterization of TBI beyond initial GCS-based classification of mild, moderate, and severe have been instituted to take the clinical presentation, imaging findings, biomarkers, and modifying host factors into account^35,36^. Such classifications may be more useful in predicting risk of complications such as progressive brain swelling or post-traumatic seizures.

Our findings are consistent with a comparable study by Surtees et al. They retrospectively evaluated clinical and radiographic features associated with prescription of ASMs for pediatric TBI. Among 167 children with TBI, 44 received ASM prophylaxis, including patients with mild, moderate, and severe TBI. In keeping with our study results, children who received ASM prophylaxis were more likely to undergo EEG monitoring and to have seizures. Abnormal imaging and neurosurgical intervention were also associated with greater likelihood of ASM prophylaxis^32^. Our study adds variables that have not been previously explored in the pediatric TBI literature. We felt it pertinent to include the admitting service as there can be sub-specialty and cultural biases regarding use of medications that are more commonly prescribed by neurologists and neurosurgeons. We also evaluated the association with EEG findings, whereas prior studies tend to report on absence or presence of EEG testing, but do not describe the results^14,32^. This is relevant as certain epileptiform abnormalities are more associated with early seizures, making these patients at higher risk and more in need of seizure prophylaxis^37,38^.

Regarding duration of ASM, a study by Amin et al evaluated duration of ASM prescription for seizure prophylaxis in adult TBI and found that there was significant variability in duration of ASM use. Of the 137 total ICU patients, 3 had seizures during their hospital admission. Most patients either received levetiracetam for less than 7 days (n = 52) or greater than 7 days (n = 66), as opposed to the typical recommendation of 7 days (n = 19)^34^. These results, in combination with our findings, highlight the lack of standardization and need for more specific recommendations to avoid risk of prescribing ASMs when they are not clinically indicated.

Limitations of our study include the retrospective nature, relying on documentation in electronic medical records and chart review, which are affected by a lack of standardization and inconsistent protocols of documentation. We used hospitalization ICD-10 codes to identify patients based on their hospitalization diagnosis, which may result in information bias and may miss certain patients that were not coded appropriately. Manual chart review was performed for all the patients included in this study, which is also at risk for possible user error. This study was based in a multi-center setting, allowing for some variance in practice, but also limited by being restricted to one geographical city (Boston, MA), which could impact its generalizability. Our data includes admissions from 2016 to 2024, an 8-year period which could include changes in neurocritical care, that may have influenced ASM prophylaxis practices. We did compare ASM prescription pre-2020 and post-2020 (accounting for the release of the 2019 Brain Trauma Foundation Pediatric guidelines) and did not find any significant differences in ASM prescription practices across these time periods (Supplementary material- Table 1).

## Conclusions

In this retrospective cohort of critically ill pediatric patients with TBI, neuroimaging abnormalities, seizures, and admission to neurology or neurosurgery services were associated with a greater likelihood of ASM prescription during hospitalization and at discharge. These findings provide insight into contemporary prescribing practices and highlight the clinical factors that may influence decisions regarding seizure prophylaxis in pediatric TBI. Future multicenter studies with larger cohorts are needed to better define the patient specific, imaging, and EEG-related characteristics associated with ASM use. Improved understanding of prescribing patterns and risk stratification may ultimately help optimize ASM use while minimizing unnecessary medication exposure and potential adverse effects.

## Supplementary Material

This is a list of supplementary files associated with this preprint. Click to download.

- SuppFigure1.jpeg
- Table1.jpg
- Table2.jpg
- SuppTable1.jpg
- SuppTable2.jpg

Table 1 and 2 are available in the Supplementary Files section.
